# Prospects for Emerging Infections in East and Southeast Asia 10 Years after Severe Acute Respiratory Syndrome

**DOI:** 10.3201/eid1906.121783

**Published:** 2013-06

**Authors:** Peter W, Horby, Dirk Pfeiffer, Hitoshi Oshitani

**Affiliations:** Oxford University Clinical Research Unit, Hanoi, Vietnam (P.W. Horby); University of Oxford, Oxford, UK (P.W. Horby);; National University of Singapore (P.W. Horby); Singapore;; Royal Veterinary College, London, UK (D. Pfeiffer);; Tohoku University Graduate School of Medicine, Sendai, Japan (H. Oshitani)

**Keywords:** emerging infections, East Asia, Southeast Asia, SARS, severe acute respiratory syndrome, influenza A(H5N1), animal health, viruses, bacteria, influenza, respiratory infections

## Abstract

It is 10 years since severe acute respiratory syndrome (SARS) emerged, and East and Southeast Asia retain a reputation as a hot spot of emerging infectious diseases. The region is certainly a hot spot of socioeconomic and environmental change, and although some changes (e.g., urbanization and agricultural intensification) may reduce the probability of emerging infectious diseases, the effect of any individual emergence event may be increased by the greater concentration and connectivity of livestock, persons, and products. The region is now better able to detect and respond to emerging infectious diseases than it was a decade ago, but the tools and methods to produce sufficiently refined assessments of the risks of disease emergence are still lacking. Given the continued scale and pace of change in East and Southeast Asia, it is vital that capabilities for predicting, identifying, and controlling biologic threats do not stagnate as the memory of SARS fades.

It is a decade since severe acute respiratory syndrome (SARS) emerged and in a few dramatic months redefined perceptions of global vulnerability to emerging infectious diseases. It is believed that SARS originated from southern China, with the first cases identified in Guangdong Province, China, where sporadic cases and small outbreaks occurred between November 2002 and early January 2003. A larger outbreak, triggered by nosocomial transmissions in 2 hospitals, began during mid-January 2003 in Guangzhou city, the capital of Guangdong Province (*1*). On February 11, 2003, the international community, including the World Health Organization (WHO), became aware of this unusual cluster of severe pneumonia cases, which included many health care workers. Detailed information about the outbreak was not available to the international community, and when WHO issued a global alert on March 12, 2003, the virus had already spread to other countries and caused outbreaks in areas outside Guangdong, including Hong Kong, China; Hanoi, Vietnam; Singapore; and Toronto, Ontario, Canada.

The SARS epidemic provided a dramatic demonstration of the weaknesses in national and global capacities to detect and respond to emerging infectious diseases, and it was in many ways a watershed event that had a transformative effect on many of the clinical, public health, and other professionals involved. But has the response to SARS had any lasting effect on the probability of new infectious agents emerging, being detected at an early stage of emergence, and being effectively controlled?

More than 30% of the global population lives in East and Southeast Asia, and despite impressive improvements in health, infectious diseases remain a major problem in the region. In 2010, 47% of the estimated 2.1 million deaths among children <5 years of age in Southeast Asia were attributable to infectious diseases (e.g., pneumonia and acute diarrhea) (*2*). Alongside this existing pool of known human pathogens, a large and diverse population of mammalian wildlife species and domestic livestock reside in the region, acting as reservoirs or amplifying species from which new infectious diseases of humans might emerge (*3*,*4*). The reemergence of highly pathogenic avian influenza A(H5N1) virus in 2004, the isolation of novel bat-associated reoviruses from humans in Malaysia in 2006, and the discovery of a novel tick-borne bunyavirus associated with fever and thrombocytopenia in rural farmers in China in 2009 attest to the existence of a pool of potential zoonotic pathogens in East and Southeast Asia (Table) (*8**,**9**,**12*). We review how the conditions that drive the emergence of infectious diseases and the systems to detect and control them have changed in East and Southeast Asia in the decade since SARS.

**Table:** Emerging and reemerging infectious disease events detected in East and Southeast Asia, 2004–2011*

Year, pathogen	Pathogen type	Driver of resistance	Location	Ref
2004				
Influenza A(H5N1)	Orthomyxovirus	Agricultural industry changes	East and Southeast Asia	(*5)*
2005				
*Streptococcus suis* serotype 2	Bacteria	Agricultural industry changes/co-infection with porcine reproductive and respiratory syndrome virus	China/Vietnam	(*6,7)*
2006				
Melaka virus	Reovirus	Improved detection	Malaysia	(*8*)
Kampar virus	Reovirus	Improved detection	Malaysia	(*9*)
2009				
Artemisinin-resistant malaria	Protozoa	Artemisinin or artesunate monotherapy	Cambodia	(*10*)
Reston Ebola virus†	Filovirus	Improved detection/co-infection with porcine reproductive and respiratory syndrome virus	Philippines	(*11*)
Severe fever with thrombocytopenia syndrome	Bunyavirus	Unknown	China	(*1**2*)

*Ref, reference. †Detected in swine but not shown to cause human disease.

## Altered Ecosystems

**Figure 1:**
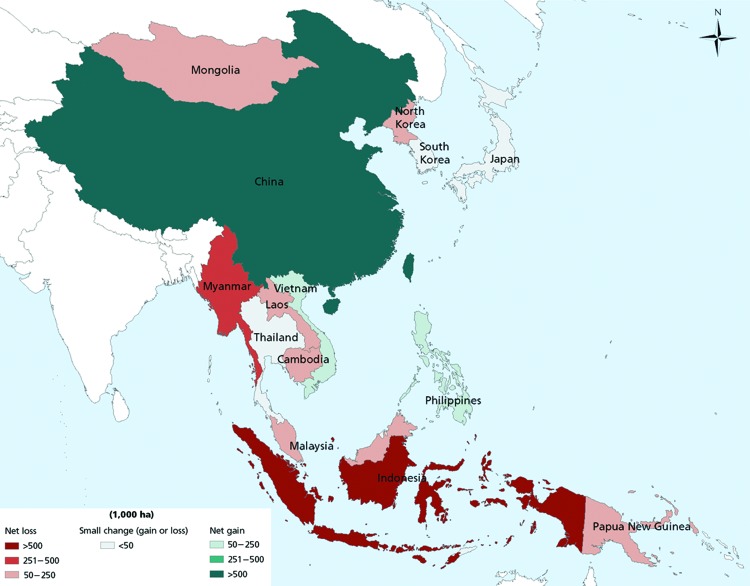
Annual change in forest area by country, 2005–2010. Source: Food and Agriculture Organization of the United Nations Global Forest Resources Assessment 2010 (www.fao.org/forestry/fra/fra2010/en/).

Over the past decade, East and Southeast Asia have been home to many of the top-performing world economies, and this macroeconomic success has resulted in large increases in the demand for natural resources. The demand for hardwood, firewood, wood pulp, agricultural and grazing land, living space, roads, minerals, and power has had an enormous effect on the ecosystems of the region. Deforestation occurred throughout the 1990s, but the last decade has seen net increases in forested areas of China, the Philippines, and Vietnam because of active afforestation (including new commercial plantations). Net forest losses continue, however, in Myanmar, Cambodia, Indonesia, and Papua New Guinea (Figure 1) (*13*).

The conversion of natural environments into agricultural or other commercially viable land (e.g., dams, mines) is usually associated with a decrease in biodiversity. A reduction in biodiversity can lead to increased disease transmission through a variety of mechanisms (e.g., reduced predation and competition) and cause an increase in the abundance of competent hosts and the loss of buffering species, leading to increased contact between amplifying host species and compatible pathogens (*14*). Although a reduction in biodiversity can lead to increased disease transmission, a large diversity of mammalian wildlife species is also associated with a large diversity of microbial species, which both increase toward the equator (*3*,*4*). Therefore, tropical areas (e.g., Myanmar, Cambodia, and parts of Indonesia) that have a rich pool of existing and potential pathogens but are experiencing ongoing ecosystem disruption and biodiversity loss may be at a particularly high risk for the emergence of zoonotic diseases.

**Figure 2:**
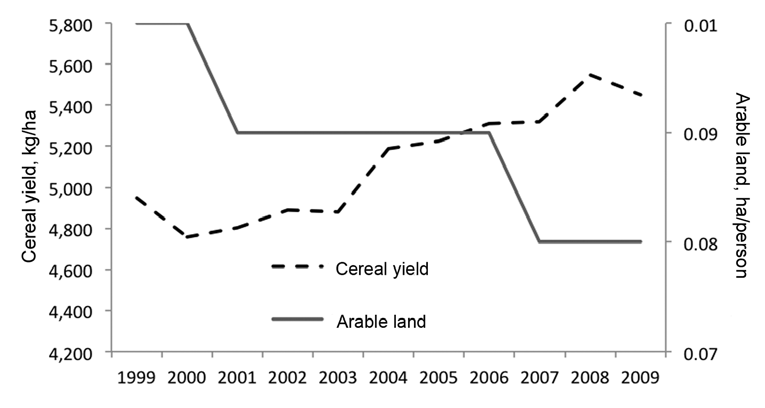
China’s cereal production yield and arable land area, 1999–2009. Source: The World Bank Agriculture and Rural Development (http://data.worldbank.org/topic/agriculture-and-rural-development).

Land-use changes are ongoing, but much of East and Southeast Asia already has very high pressures on productive land. The rate of land-use change in much of the region has probably peaked, and the region is now in an era of increasing intensification of land productivity. In fact, over the last decade, China has increased agricultural output despite a slight decrease in total agricultural land area (Figure 2) (*15*). This intensification is driven largely by demographic pressures, which are predicted to result in a 70% increase in food production by 2050; the consumption of grains is expected to decrease and demand for meats, fruits, and vegetables is expected to increase (*15*). The recent high and volatile prices for food commodities are a good indicator of the current vulnerability of agricultural production systems.

The environmental consequences of intensified agricultural production include the depletion and degradation of river and groundwater, reduced soil quality, and water and soil contamination with chemical fertilizers and pesticides. The loading of aquatic ecosystems with nitrogen and phosphorous (eutrophication) is a widespread environmental change with an as-yet unquantified effect on the risk for disease emergence. Eutrophication can result in potentially harmful blooms of cyanobacteria, but little is known about the effect on pathogens that cause disease in animals and humans. There is, however, evidence that eutrophication can alter ecosystems in such a way as to increase the transmission of parasitic diseases of amphibians, the concentration of *Vibrio cholerae*, and the abundance of mosquito vectors (*16*). Given the trend of increasing intensification of crop and animal production in East and Southeast Asia, much more attention should be given to the effect of the large-scale contamination of water and soil with nitrogen, phosphorous, and other chemicals on the functioning of ecosystems and on disease dynamics.

## Livestock Production

Demand for livestock products in East and Southeast Asia has risen dramatically over the past 50 years: the per capita consumption of meat in developing countries has more than tripled since the early 1960s, and egg consumption has increased 5-fold (*17*). The increased demand for meat has been met by more intensive and geographically concentrated production of livestock, especially pigs and poultry. East and Southeast Asia are home to 569 million pigs (60% of the world pig population) and 9.2 billion poultry (43% of the world poultry population) (*18*). The Food and Agriculture Organization of the United Nations (FAO) has projected that pork consumption in China will increase by 55% between 2000 and 2030 (*19*), and Rabobank, an agricultural finance group, predicts a 45% increase in global meat demand over the next 20 years, with 70% of that occurring in Asia. The demand will be particularly strong for poultry because of its relatively low cost and short production cycle, and because there are fewer cultural restrictions regarding poultry than there are that concern pork and beef.

In addition, meat-producing companies will continue to consolidate at the global level. The intensification of livestock farming often results in more effective separation of domestic and wild animals, improved veterinary supervision and input, reduced movement of animals, and reduced species mixing, all of which may reduce the likelihood of disease emergence. However, higher densities of short production–cycle domestic animals, such as pigs and, in particular, poultry, introduce a vulnerability because such animals usually have limited genetic variation. Higher genetic diversity within a host species is often associated with differences in susceptibility to infection, thereby limiting the potential for infections to spread rapidly (*20*). Recent outbreaks of highly pathogenic porcine reproductive and respiratory syndrome virus throughout East and Southeast Asia, which at times co-occurred with outbreaks of *Streptococcus suis* infections, and the detection of Reston Ebola virus infection in pigs in the Philippines highlight the ongoing risk for disease emergence, amplification, and crossover from livestock to humans in East and Southeast Asia (*6*,*11*).

In East and Southeast Asia, antimicrobial drugs are used extensively in the livestock and aquaculture sectors to treat or prevent infections, and they are used non-therapeutically as growth promoters, which requires the prolonged administration of sub-therapeutic doses. This practice has a demonstrable effect on the emergence and prevalence of potentially clinically relevant resistant microorganisms in food animals. Furthermore, the subsequent excretion of antimicrobial drugs into the environment may subject environmental bacteria to antimicrobial selection pressures (*2**1*). It is clear that the continued use of non-therapeutic antimicrobial drugs in livestock and aquaculture industries that are increasing in scale and intensity poses a threat to human and animal health (*22*).

## Wildlife and Farm Biosecurity

Reducing contact between domestic and wild animals, whether the wild animals remain wild or are captive in breeding farms or markets, is a key tactic recommended by the FAO for reducing risk to human health, and this reduced contact is part of the wider FAO strategy for biosecurity. Improving biosecurity in farms in East and Southeast Asia is a major challenge because a large proportion of the farming is done in backyard and small- to medium-scale commercial farms, and there is often a mix of commercial and backyard farming in any 1 location (*23*). The longer-term vision is to restructure the livestock production sector toward a more integrated and controlled system in which controls benefit animal health and welfare, human health, and commercial profitability without adversely affecting the livelihood of poor persons.

In East and Southeast Asia, increasing intensification of animal husbandry may lead to healthier, better isolated animals and a subsequent lowered risk for emerging disease events. However, should an emerging infectious disease event occur, this intensification may result in greater amplification of disease in large, naive monocultures, as demonstrated in the Netherlands when they experienced major outbreaks of classical swine fever in 1997–1998 and avian influenza in 2003. The role of civet cats in the SARS pandemic and the smuggling of avian influenza A(H5N1) virus–infected birds of prey into Europe showed that the legal and illegal wildlife trade is an effective conduit for zoonotic pathogens to enter new niches (*24*,*25*). Wild animal products remain popular in East and Southeast Asia as traditional medicines, tonics, food delicacies, or symbols of wealth. Although all 10 countries in the Association of Southeast Asian Nations (ASEAN) are signatories to the Convention on International Trade in Endangered Species of Wild Fauna and Flora, Asia continues to host the largest illegal wildlife trade in the world (*26*).

## Travel and Trade

The regional and global connectivity of East and Southeast Asia continues to increase; there is visa-free travel between ASEAN countries, relaxation of previously restrictive international travel policies in some countries, and a proliferation of budget airlines. Increased travel and trade between Africa and Asia is a particularly notable phenomenon: exports from Africa to Asia increased 20% from 2003 to 2008. This Africa–Asia traffic is a new corridor for the exchange of potential emerging infectious pathogens.

The ongoing development of a regional road transport network within East and Southeast Asia will also offer new opportunities for pathogen dispersal because, compared with air travel, roads offer a more egalitarian form of connectivity that includes animals as well as humans. Increases in domestic travel also continue; only 10% of the world is now classified as remote (i.e., >48 hours travel time to a big city), and an estimated 2.5 billion passenger trips were made during China’s 2011 Lunar New Year celebrations—the greatest annual human migration on earth. Increased connectivity provides greater opportunities for pathogens to disperse beyond their traditional niches and presents a formidable challenge to the tracking and containment of outbreaks (*27*).

## Urbanization, Human Demographics, and Behavior

Between 2011 and 2050, the global population is expected to increase from 3.6 billion to 6.3 billion (an increase of 2.6 billion [72%]), and this increase will be concentrated in cities (*28*). Most of the growth will occur in developing countries, particularly in Asia, which will experience an increase in its urban population of 1.4 billion persons. In many ways, this concentration of population growth in urban areas is a positive development, and the popularity of cities is a testament to the fact that cities generally provide better economic and education opportunities, better living and sanitation conditions, better nutrition, and therefore better health than in underdeveloped rural areas.

Cities are, however, key in the epidemiology of many infectious diseases because they can function as “pace-makers” that drive temporal and spatial transmission dynamics of local epidemiology (e.g., dengue), hubs for national and global spread (e.g., SARS and HIV), or bridges between human and animal ecosystems (e.g., influenza A[H5N1]). East and Southeast Asia have made considerable progress in health and social welfare improvements: during 2000–2010, a total of 125 million persons in China and India moved out of slum conditions. However, urban poverty remains a concern. In 2010, an estimated 500 million persons in Asia lived in slums (*29*), and, at the end of 2008, there were an estimated 140 million rural migrant workers in China, many of whom lacked residency rights and had limited access to health care and other social supports (*30*). Circular migration between rural and urban settings is common and may facilitate the transfer of pathogens from wild or rural ecosystems to urban areas, with the potential for rapid amplification in settings with high concentrations of migrant workers.

## Health Systems

Almost 35% of the emerging infectious diseases identified in Asia during 1940–2004 represent the emergence of a new pattern of antimicrobial drug resistance (*4*). The major driver of such resistance is drug pressure. In East and Southeast Asia, the sequential development of resistance by malaria parasites to chloroquine, sulfadoxine-pyrimethamine, mefloquine, and now artemisinin is a measure of both the adaptive capacity of the parasite and the failure of health systems to implement effective drug combination and cycling strategies to avoid resistance. Bacteria in East and Southeast Asia show high rates of resistance to antimicrobial agents; examples include multidrug-resistant *Acinetobacter baumannii*, *Salmonella enterica*, and *Enterobacteriaceae* (*31*). A high level of antimicrobial resistance is a marker of the failure to control access to antimicrobial drugs and to influence prescribing behaviors. Over-the-counter antimicrobial drugs are available without a prescription throughout much of East and Southeast Asia, even though antimicrobial drugs are officially prescription-only medicines in most countries. Left unchecked, the supply- and demand-side incentives for inappropriate antimicrobial drug use will lead to a region awash with antimicrobial drugs and with the potential for public health disasters, such as artemisinin resistance, which now threatens global malaria control.

## Surveillance and Response

**Figure 3:**
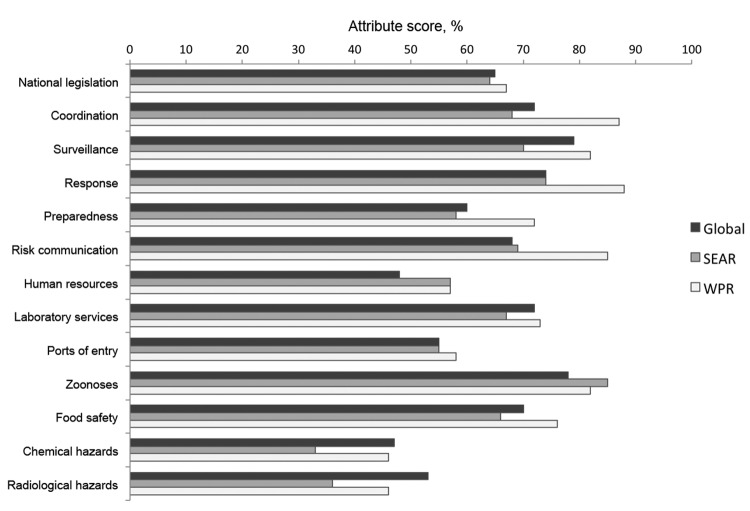
Self-reported global and regional average attribute scores for international health regulations core capacities, 2011. Source: World Health Organization, Summary of 2011 States Parties Report on International Health Regulations Core Capacity Implementation. (www.who.int/ihr/publications/WHO_HSE_GCR_2012.10eng/en/index.html). The attribute score is the percentage of attributes (a set of elements or functions that reflect the level of performance or achievement of an indicator) in which moderate or strong technical capacity has been attained in each core capacity area. SEAR results are the average for 11/11 eligible countries. WPR results are the average for 19/27 eligible countries (8 countries did not complete the questionnaire in 2011). SEAR, World Health Organization’s (WHO) South-East Asia Region; WPR, WHO Western Pacific Region.

The SARS pandemic highlighted what had been apparent to some since the 1990s: few countries possessed the necessary surveillance and response capacities to rapidly detect and control emerging infectious diseases (*32*). The deficiencies of the 1969 International Health Regulations at the global level had long been recognized, and attempts to revise them were ongoing before 2003, but the SARS outbreak added new urgency and momentum for change. The International Health Regulations were successfully revised in 2005, and for the first time they defined a series of core capacities that each country is required to establish to detect, report, and control public health emergencies of international concern. The target for attaining these core capacities was set as June 2012, and an assessment undertaken in 2011 found that although these core capacities had not yet been fully achieved in several countries of East and Southeast Asia, considerable progress had been made (Figure 3) (*33*). For example, the influenza surveillance network in China expanded from 63 laboratories and 197 sentinel hospitals in 2005 to 441 laboratories and 556 sentinel hospitals in 2009 (*34*). Field epidemiology training programs have played a central role in strengthening epidemiology capacity in human and animal health, and new field epidemiology training programs were implemented in Laos, Mongolia, and Vietnam in 2009 and in Cambodia in 2011 (*35*). An analysis of the global capacity for detecting outbreaks showed improvements in the median time from outbreak start to outbreak discovery between 1996 (29.5 days) and 2009 (13.5 days) and from start to public communication (40 days in 1996 to 19 days in 2009); the WHO Western Pacific Region was the only WHO region that showed a statistically significant improvement in both areas (*36*).

Many of these improvements were facilitated by a large increase in political and financial support for emerging infectious disease surveillance and response from national governments and donor agencies after the outbreaks of SARS and influenza A(H5N1). Although data on the total expenditure for emerging infectious disease surveillance, preparedness, and response in East and Southeast Asia are not available, examples of international support include the first and second Asian Development Bank Greater Mekong Subregion Regional Communicable Diseases Control Projects ($38.75 million and $49 million, respectively), the Canada–Asia Regional Emerging Infectious Disease Project ($4.3 million), the US Government foreign assistance for disease control, research, and training (>$500 million in Asia during 2004–2011), and the US Agency for International Development’s Pandemic and Emerging Threats Program. As a consequence, pandemic and epidemic preparedness planning has improved in most countries of East and Southeast Asia, but gaps between the plans and the ability to operationalize them remain in many countries (*37*,*38*).

Since 2003, international and national authorities have increasingly recognized the importance of more effective animal health surveillance. However, limited resources in most countries have meant that investments into improved surveillance capacity have occurred largely in those countries affected by major outbreaks, such as the case with an outbreak of Nipah virus infection in Malaysia and of influenza A(H5N1) virus infection in Thailand, China, Vietnam, and Indonesia. Where these types of investments did occur, they were often species- and threat-specific, rather than to facilitate strategic enhancement of generic surveillance efforts for dealing with emerging disease threats. Not too dissimilar from the situation in high income countries, institutional and administrative boundaries between the human and animal health sectors have largely prevented the development of integrated surveillance systems.

## Regional and International Partnerships

It has been said that there have been more international meetings about influenza A(H5N1) virus than human cases of the disease. Whether this is true or not, a benefit of the many meetings held after the outbreaks of SARS and influenza A(H5N1) virus has been a strengthening of regional and international professional partnerships. Clinicians, epidemiologists, virologists, veterinarians, and public health officials in East and Southeast Asia are now better connected and familiar with their colleagues than they were before 2003: examples include the South East Asia Infectious Disease Clinical Research Network, the Mekong-Basin Disease Surveillance System, increasing membership of East and Southeast Asia countries in the Training Programs in Epidemiology and Public Health Interventions Network , and the establishment in 2003 of the ASEAN+3 Expert Working Group on Communicable Diseases (*39**,**40*). Networks of trusted colleagues are a powerful force for sharing expertise, clearing confusion, and bridging divides, and these newly formed partnerships are perhaps one of the greatest unquantified achievements of the last decade.

To coordinate and harmonize the diversity of initiatives spawned by SARS, the WHO South East Asia Office and Western Pacific Office jointly developed the Asia Pacific Strategy for Emerging Diseases in 2005. This plan provides a common framework for strengthening national and regional surveillance and response capacity for emerging infectious diseases in the 48 countries of the Asia Pacific Region; it was revised and re-endorsed by the WHO Regional Committees in 2010.

A more troublesome dimension of international partnerships since 2003 has been the dispute over the sovereignty and sharing of pathogen samples. Although these disputes have not benefited disease surveillance in the short term, they do have a legitimate basis, and it must be hoped that in the medium term, an airing and resolution of these issues will result in greater trust, improved surveillance, and a more equitable distribution of benefits. In this context, the ratification in 2011 of WHO’s Pandemic Influenza Preparedness Framework for the Sharing of Influenza Viruses and Access to Vaccines and Other Benefits is a major step forward.

## Conclusions

A major shift, from West to East, is underway in the global center of gravity: East and Southeast Asia are becoming the dominant force of economic, social, and environmental change. While rapid development has brought East and Southeast Asia many benefits, it has also resulted in widening health inequalities, environmental degradation, increased migration and urbanization, and a concentration of persons, food production, and economic activity. These changes might facilitate the emergence and transmission of new pathogens, but it would be simplistic and disingenuous to present the extensive changes in East and Southeast Asia as inevitably increasing the risk of emerging infectious diseases. It seems likely that the probability of new emerging infections may be reduced by many of these socioeconomic changes, such as urbanization and the industrialization and commercialization of agriculture and food production; however, the scale and effect of any individual emergence event may increase because of a greater concentration and connectivity of livestock and persons. Surveillance and response capacities have improved in the last decade, and East and Southeast Asia are far better prepared to detect and respond to emerging infectious diseases. However, we are still lacking the tools and methodologies to produce a sufficiently refined assessment of the distribution and profile of disease emergence risks that encompasses geographic heterogeneity; the interaction of different drivers of pathogen evolution, crossover, and dispersion; and dynamic systems and the uncertainty inherent in such assessments. Given the continued scale and pace of change in East and Southeast Asia, it is vital that the capacity to predict and identify biologic threats and to protect the public’s health does not stagnate as the memory of SARS fades.
